# The anodal tDCS over the left posterior parietal cortex enhances attention toward a focus word in a sentence

**DOI:** 10.3389/fnhum.2014.00992

**Published:** 2014-12-09

**Authors:** Takehiro Minamoto, Miyuki Azuma, Ken Yaoi, Aoi Ashizuka, Tastuya Mima, Mariko Osaka, Hidenao Fukuyama, Naoyuki Osaka

**Affiliations:** ^1^Department of Advanced Human Sciences, Graduate School of Human Sciences, Osaka UniversityOsaka, Japan; ^2^Department of Psychology, Graduate School of Letters, Kyoto UniversityKyoto, Japan; ^3^Human Brain Research Center, Graduate School of Medicine, Kyoto UniversityKyoto, Japan

**Keywords:** posterior parietal cortex, tDCS, attention, working memory, reading span test

## Abstract

The posterior parietal cortex (PPC) has two attentional functions: top-down attentional control and stimulus-driven attentional processing. Using the focused version of the reading span test (RST), in which the target word to be remembered is the critical word for comprehending a sentence (focused word) or a non-focused word, we examined the effect of tDCS on resolution of distractor interference by the focused word in the non-focus condition (top-down attentional control) and on augmented/shrunk attentional capture by the focused word in both the focus and non-focus conditions (stimulus-driven attentional processing). Participants were divided into two groups: anodal tDCS (atDCS) and cathodal tDCS (ctDCS). Online stimulation was given while participants performed the RST. A *post-hoc* recognition task was also administered in which three kinds of words were presented: target words in the RST, distractor words in the RST, and novel words. atDCS augmented the effect of the focused word by increasing differences in performance between the focus and non-focus conditions. Such an effect was not observed in the ctDCS group. As for the recognition task, atDCS again produced the augmented effect of the focused words in the distractor recognition. On the other hand, ctDCS brought less recognition of non-focused target words in comparison to sham. The results indicate that atDCS promotes stimulus-driven attentional processing, possibly by affecting neural firing in the inferior parietal regions. In contrast, ctDCS appears to prevent retrieval of less important information from episodic memory, which may require top-down attentional processing.

## Introduction

Transcranial direct current stimulation (tDCS) modulates a variety of psychological processes such as motor functions and cognitive control (Fregni et al., 2005, 2006; Marshall et al., 2005; Stagg et al., 2009) by enhancing or suppressing the resting membrane potential. This technique is considered a candidate for the neuro-rehabilitation of neurological or cognitive deficits, as it encourages neuro-plasticity (Paulus, 2011; Vallar and Bolognini, 2011). In fact, tDCS was reported to improve speech performance and relearning when it was administered to the stroke-damaged left hemisphere of chronic aphasic patients (Holland and Crinion, 2012). Other studies have shown that tDCS over the dorsolateral prefrontal cortex enhanced working memory (Fregni et al., 2005), which is a platform for a large number of higher order cognitive processes.

The posterior parietal cortex (PPC) plays a critical role in attentional processing. It is well known that parietal-dependent attentional processing had two divisions: top-down attentional control and stimulus-driven attentional reorientation. Top-down attentional control relies on the superior part of the PPC, which includes the intraparietal sulcus (Corbetta and Shulman, 2002). For example, covert attention toward an instructed spatial location produced sustained activation of the intraparietal sulcus (Corbetta et al., 2000). On the other hand, stimulus-driven attention reorientation depends on the temporoparietal junction (TPJ), which consists of the inferior part of the PPC and the superior part of the temporal cortex (Corbetta and Shulman, 2002). Activation of the TPJ was found when attention was captured by salient information or when attention was reoriented toward spatially unexpected areas (Corbetta et al., 2000). Those results indicate that the TPJ activates in response to environmentally salient information. Recent studies have reported that the salient information has to be task-relevant in order to activate the TPJ (Geng and Mangun, 2011). In fact, when a sensory stimulus that has high saliency was completely irrelevant, activation of the TPJ disappeared (Geng and Mangun, 2009). Therefore, the TPJ is thought to activate in response to environmentally-salient information that is relevant to the current goal in order to reorient attention toward the information.

In the visuospatial domain, anodal tDCS over the PPC enhances visuospatial attention on the contralateral side, while cathodal tDCS enhanced attention on the ipsilateral side (Sparing et al., 2009), using a simple visuospatial detection task (Hilgetag et al., 2001).

**Figure 1:**
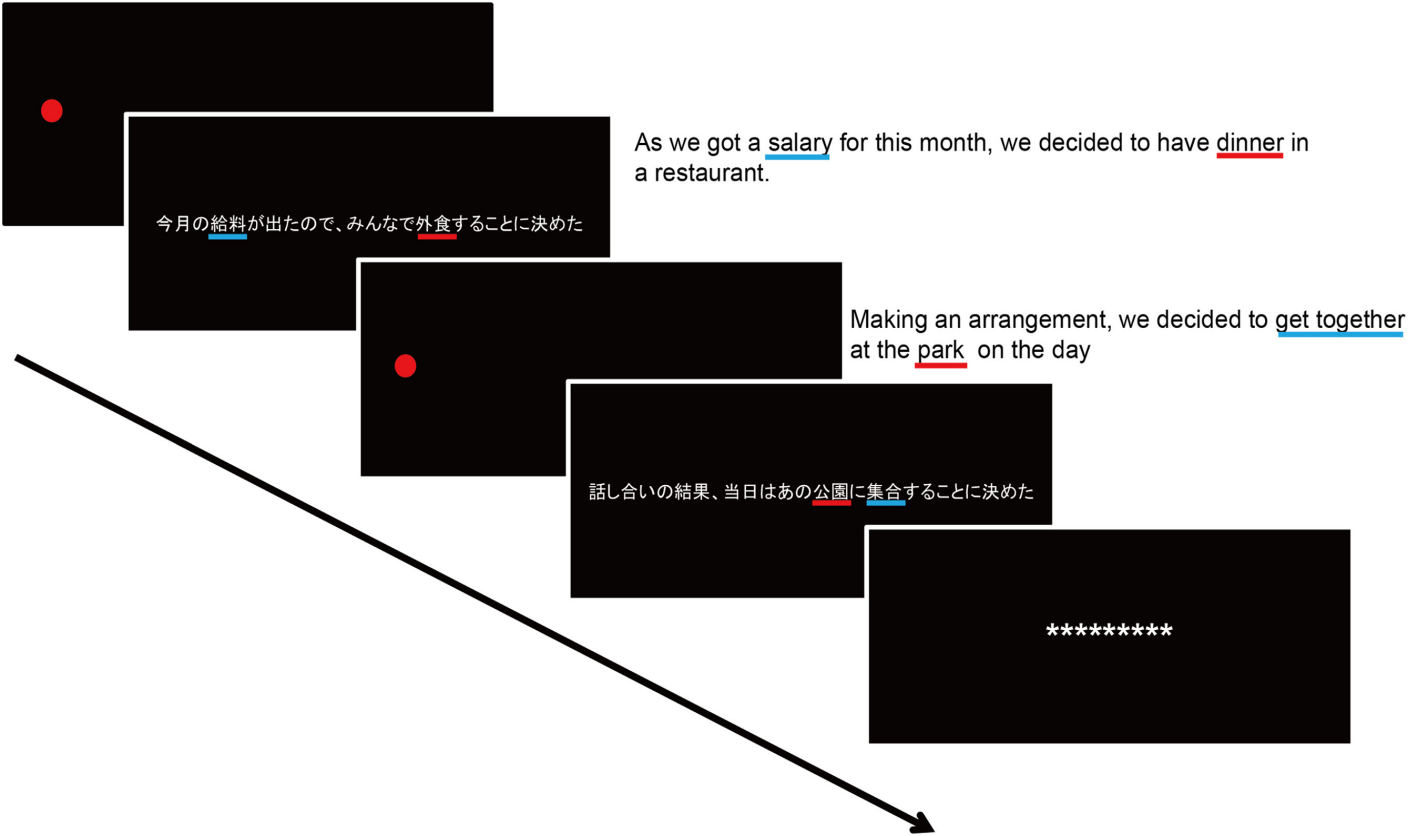
**A schematic diagram of the reading span task**. A red fixation circle was presented at the beginning of the task, which was followed by a sentence in which a target word was underscore by a red line. Participants were instructed to read a sentence aloud and to remember the target word. After presentation of four sentences, a recall screen was given, and participants reported the words they remembered. For a clarification purpose, focused words were underlined by red lines and non-focused words were done by the blue ones. English translations were given next to the Japanese sentences.

Focusing on the verbal domain, we investigated how tDCS over the PPC affects attentional processing. Specifically, we tested whether tDCS over the left PPC modulates top-down voluntary attentional control or attentional reorientation toward goal-relevant salient information while subjects processed verbal material. In order to test the effect of tDCS, we used a focused version of the reading span test (RST) (Osaka et al., 2002b, 2007). The RST requires participants to read a sentence aloud while remembering a target word underscored by a red line (Figure 1). Importantly, we manipulated the nature of the target word. In the focus condition, the target word was the focused word, meaning it is critical for comprehending the given sentence. On the other hand, in the non-focus condition, the target word was not the focused word. This condition required participants ignore the focused word for better performance. In the past, participants in this condition have falsely reported focused words as targets (Osaka et al., 2002b), indicating that focused words attract attention with importance for sentence comprehension. Thus, focused words can be conceived as salient stimuli that reorient participants' attention. Therefore, the focused version of the RST recruits two attentional processes. Top-down attentional control is demanded in order to ignore the focused word in the non-focus condition, while stimulus-driven attentional processing toward the focused word is recruited in both the focus and non-focus conditions. Applying tDCS over the left PPC, the present study examined which attentional processing is modulated by the stimulation. The left side of the PPC was selected, because we previously found greater activation in the non-focus condition than the focus condition (Osaka et al., 2007). If top-down attentional processing is modulated, promoted cortical excitability by atDCS will enhance performance in the non-focus condition with efficient cognitive control that allows participants to ignore distracting focused words compared with the sham condition, and suppressed cortical excitability by ctDCS will worsen task performance. On the other hand, if tDCS modulates stimulus-driven attentional processing, atDCS will enhance performance in the focus condition but decrease it in the non-focus condition by directing attention toward the focused word, and ctDCS will produce the opposite effect.

## Materials and methods

### Participants

A total of 32 graduate and undergraduate students participated in the present study. All subjects were healthy volunteers and reported normal or corrected-to-normal vision. Participants were assigned to either atDCS (*M* = 22.56, *SD* = 3.01, 7 females) or ctDCS groups (*M* = 21.31, *SD* = 1.40, 5 females). Participants were divided into two groups due to limited number of experimental stimuli, where same stimulus could not be used twice as repetitive usage affects memory performance. As our previous study showed that performance of the focused version of the RST differed depending on individual working memory capacity (Osaka et al., 2002a), we measured individual working memory capacity prior to the tDCS experiment (at least 5 days before) by the standard version of the RST. Mean score of the anodal group was 2.88 (*SD* = 0.62) and that of the cathodal groups was 2.78 (*SD* = 0.71). The score did not differ between groups, *F*_(1, 30)_ = 0.16, *p* > 0.05.

Before an experiment, an experimenter gave the participant a detailed description of the study, and he/she provided informed consent. The study protocol was approved by the Committee of Medical Ethics of the Graduate School of Medicine, Kyoto University, Japan. Participants received payment for their participation. The current study was conducted in accordance with the 1964 Declaration of Helsinki.

### Stimulus and apparatus

#### Reading span test

The stimuli were one-hundred sets of four Japanese sentences that were used previously (Osaka et al., 2002b, 2007). Each sentence consisted of 20–30 characters and contained more than two nouns. The nouns consisted of two Chinese characters, and their mora phoneme varied from 2 to 5 in length. We also controlled frequency of the nouns, using standard Japanese word frequency values (Amano and Kondo, 2000).

As described previously (Osaka et al., 2002b, 2007), 100 undergraduate students who did not participate in the experiments selected the focus word of each sentence. The focus word was defined when more than 70% of the students selected a noun as the most important word in the sentence for understanding.

Using the focus words, we prepared two kinds of RSTs. One was the focus version of the RST (F-RST), where the target words corresponded to the focus words. The other was the non-focus version of the RST (NF-RST), where the target words were not the focus words. Therefore, target words were congruent with the focus words in the F-RST, but incongruent with the focus words in the NF-RST. Each trial consisted of four sentences (set-size of four) and a total of 12 trials were prepared for each RST with 2 practice trials.

Stimuli were presented on a 17-inch monitor located 47 cm away from the participant. A chin-rest was used to maintain visual distance.

#### Word recognition task

A total of 144 words were presented for the word recognition task in each session. One-third of the words were target words in the RSTs, and one-third were distractor words. The remaining items were novel nouns with 2 Chinese characters, and their phoneme mora ranged from 2 to 5. Stimulus presentations and log retrievals were regulated by Presentation (Neurobehavioral Systems, Inc., Albany, CA).

### tDCS

**Figure 2:**
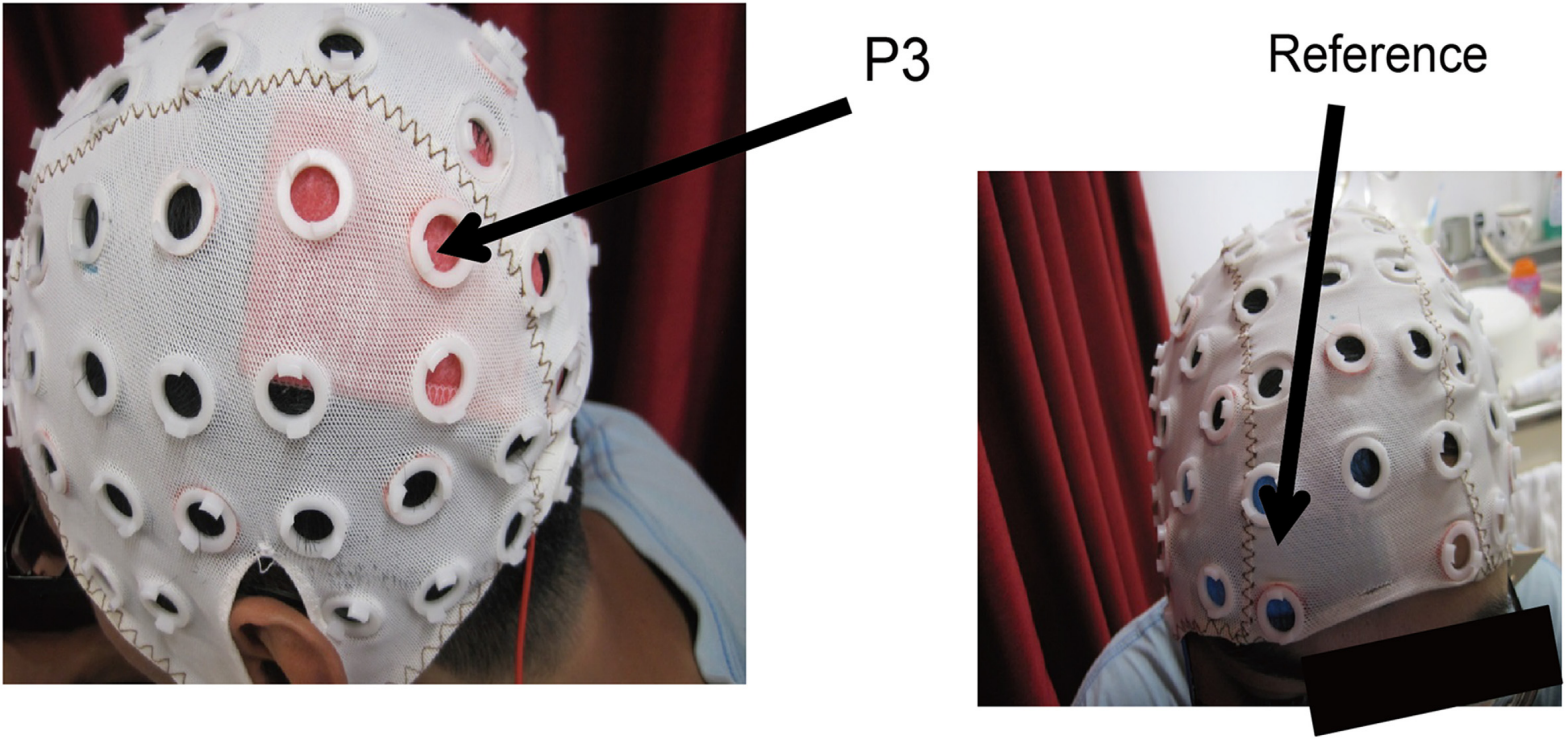
**Head locations for the electrodes**. The target region was the left posterior parietal cortex where the center of the electrodes was located at P3 in the 10–20 international measurement. The reference patch was located just above the eyebrow.

Direct current stimulation was administered to the scalp using electrodes covered with saline-soaked sponges 35 cm^2^ large (5 × 7 cm). The electrodes were connected to a battery-driven DC-stimulator Plus (NeuroConn GmbH, Ilmenau, Germany). We administered online DC stimulation (2 mA) while participants performed RSTs, and the stimulation lasted no more than 15 min. In order to activate the left PPC (atDCS), the anodal electrode was placed over P3 in accordance with the 10–20 international system (Figure 2). The cathodal electrode was attached to the contralateral supraorbital area. For hypo-stimulation (ctDCS), the positions of the electrodes were reversed from the atDCS condition. For both anodal and cathodal simulations, current faded in through 20 s and was kept stable at 2 mA during the RST. As soon as participants finished all RSTs, the experimenter terminated the stimulation. Because the RST was a self-paced task, stimulus duration varied across participants. For shams, the DC faded in through 20 s and faded out the same duration as soon as the intensity reached 2 mA.

### Experimental procedure

A schematic diagram of the RST is shown in the Figure 1. Task instructions were given at the beginning of the experiment and followed by a mount of electrodes. Two practice RST trials were given prior to the experimental trials. After the practice trials, either direct current or sham stimulation was administered. In the experimental trials, each trial began with the presentation of a red dot that was placed at the left side of the monitor for 3 s, and participants were required to fixate on it. This manipulation was included to restrict the strategy of looking at a target word before reading the sentence (Azuma et al., 2012). After the fixation, a sentence was presented, and a participant was required to read the sentence while remembering the target word underlined in red. As soon as the participant read the sentence, the experimenter clicked a mouse to present the next sentence. A recall screen was shown when the participant read four sentences, and participants reported the words they remembered. They could report the words in free order, but were not allowed to report the last target word first. A total of 12 trials were performed. Trials were equally split between the focus and non-focus conditions. The order of the conditions was counterbalanced across participants.

Immediately after the RSTs, the recognition memory test was given. In each trial, a word was presented at the center of the monitor, and participants were instructed to judge whether the word was the target in the RSTs they had just performed. They were explicitly instructed to reject the word if they noticed that the word was the distractor item; i.e., they had to reject the item when it was a distractor item or novel filler. Stimulus order (target, distractor, or filler) was randomly determined. A total of 144 trials were given.

Experiments consisted of two sessions, which were separated by a one-week washout period (Teo et al., 2011) in order to avoid a carry-over effect. The order of the brain stimulation (tDCS or sham) was counterbalanced across participants.

### Experimental design and statistical analysis

#### Reading span test

We tested factors of brain stimulation (tDCS vs. sham) and focus manipulation (focus vs. non-focus) in each tDCS group. The dependent variables were recall performance rate and the number of intrusion errors. Intrusion errors were counted when participants reported a word other than the target in a given sentence. A repeated ANOVA was performed with a statistical threshold of *p* < 0.05. Simple main effects were tested by the repeated ANOVA when a significant interaction was obtained. Because our primary goal was to test the effect of tDCS in comparison with shams, we separately analyzed data from the atDCS and ctDCS groups.

#### Recognition task

Recognition performance was computed by subtracting the yes rate for the filler from that for the target or distractor words in the sentences. Statistical analyses were separately performed on recall performance for targets and for distractors. As with the RST, we tested the effects of brain stimulation and focus manipulation in each tDCS group. Repeated ANOVAs were performed, with an alpha level of *p* < 0.05. Simple main effects were tested by the repeated ANOVA when a significant interaction was obtained.

## Results

Although our primary interest was the focus effect (i.e., an absolute value of difference between performance in the focus condition and that in the non-focus condition), we performed a within-subject ANOVA including two factors (the focus-manipulation and brain-stimulation). This procedure was taken in order to clearly describe which direction the brain stimulation affected performance in the focus and non-focus conditions (e.g., enhancement of the focus effect was due to increased performance in the focus condition or decreased performance in the non-focus condition). Figures of the focus effects were available in a Supplementary Material.

### Reading span test

#### Recall performance

**Figure 3:**
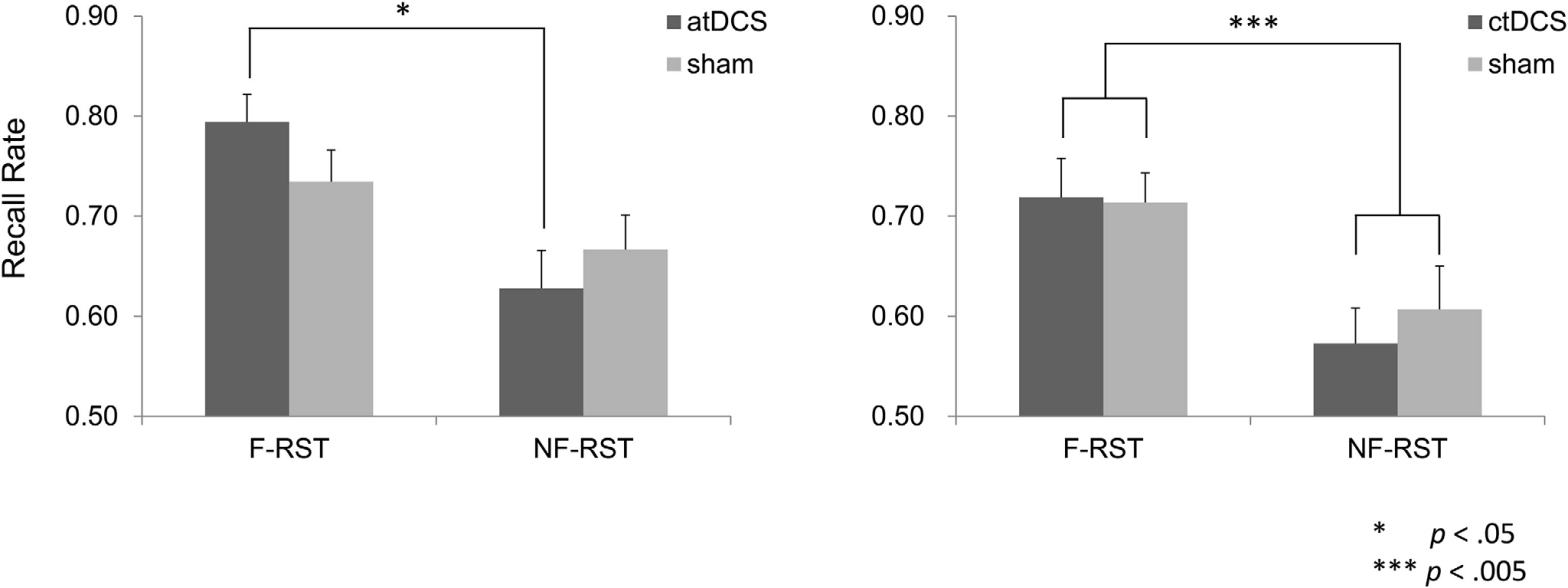
**Recall performance of the Reading Span Test (RST) in the focus and non-focus conditions**. atDCS produced augmented difference in recall performance between the focus and non-focus conditions, in comparison to the sham stimulation. Such effect was not observed in the ctDCS group.

In the atDCS group, atDCS augmented difference in recall performance between the focus and the non-focus conditions, comparing with the sham stimulation. (Figure 3). A repeated ANOVA showed a significant main effect of the focus manipulation [*F*_(1, 15)_ = 14.53, *p* < 0.005], but not of brain stimulation [*F*_(1, 15)_ = 0.16, *p* > 0.05]. In the atDCS condition, a significant interaction was obtained [*F*_(1, 15)_ = 5.45, *p* < 0.05] and *post-hoc* analysis for a simple main effect of the focus condition showed a significant effect in the DC condition [*F*_(1, 15)_ = 20.17, *p* < 0.001]. The effect was only marginal for the sham condition [*F*_(1, 15)_ = 3.22, *p* < 0.10]. Simple main effects of brain stimulation were not significant in either the focus condition [*F*_(1, 15)_ = 3.04, *p* > 0.05] or non-focus condition [*F*_(1, 15)_ = 1.30, *p* > 0.05]. Therefore, the obtained interaction was attributed to an augmented effect of the focus manipulation by the atDCS.

The above interactions were not seen in the ctDCS group. Only a main effect of the focus manipulation was obtained (Figure 3). A repeated ANOVA yielded a significant main effect of the focus manipulation [*F*_(1, 15)_ = 14.64, *p* < 0.005], but not a main effect of the brain stimulation [*F*_(1, 15)_ = 0.51, *p* > 0.05]. Nor was any interaction observed [*F*_(1, 15)_ = 0.63, *p* > 0.05].

#### Intrusion errors

**Figure 4:**
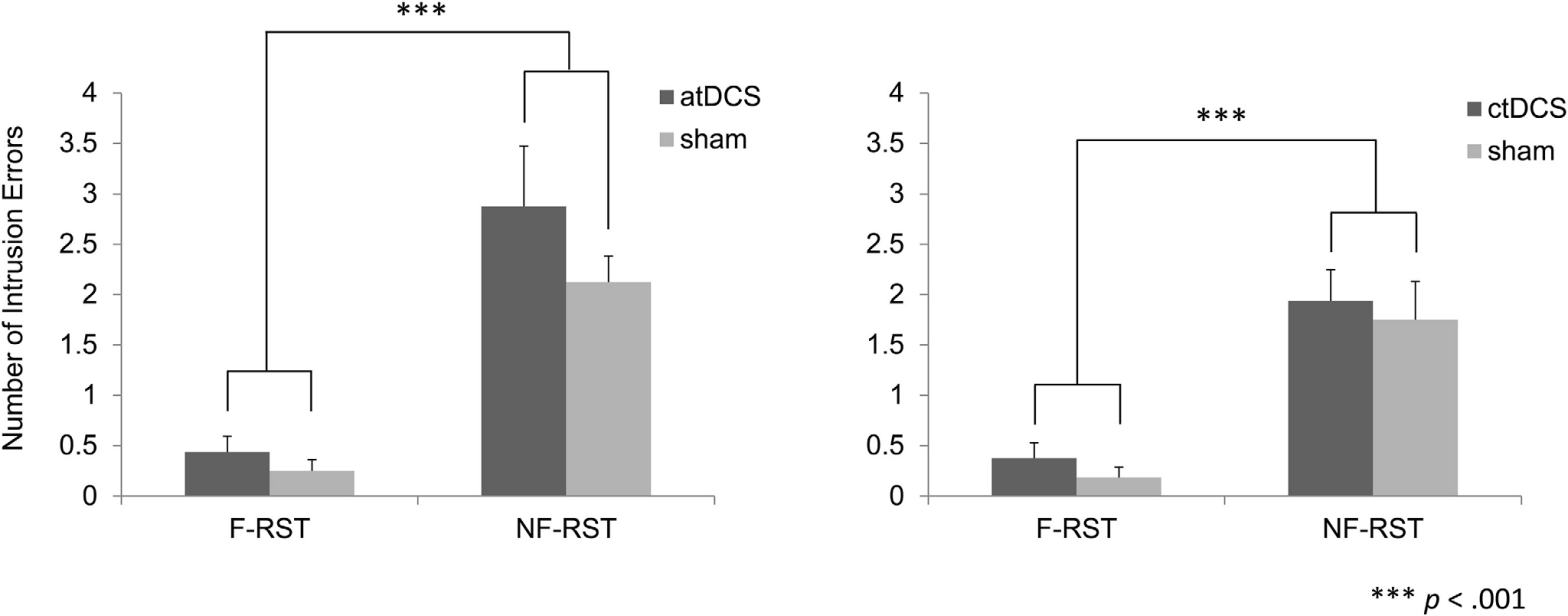
**Number of intrusion errors in the focus and non-focus conditions**. In both the atDCS and ctDCS groups, more intrusion errors were observed in the non-focus condition than the focus condition. However, brain stimulation did not modulate those errors.

In the atDCS group, the number of intrusion errors was greater in the non-focus condition than in the focus condition across all brain stimulation conditions (Figure 4). However, the number did not differ between the tDCS and sham condition. Additionally, experimental factors did not show any interaction with the errors. A repeated ANOVA showed a significant main effect of the focus manipulation [*F*_(1, 15)_ = 39.35, *p* < 0.001], but not for brain stimulation [*F*_(1, 15)_ = 2.27, *p* > 0.05]. The interaction between factors was also non-significant [*F*_(1, 15)_ = 0.81, *p* > 0.05].

Similarly, in the ctDCS condition, the number of intrusion errors was greater in the non-focus condition than in the focus condition (Figure 4). However, the number was equivalent across brain stimulation conditions. A repeated ANOVA showed a significant main effect of the focus manipulation [*F*_(1, 15)_ = 39.23, *p* < 0.001], but not for brain stimulation [*F*_(1, 15)_ = 0.48, *p* > 0.05]. The interaction between factors was not significant [*F*_(1, 15)_ < 0.01, *p* > 0.05].

### Recognition performance

#### Target recognition

**Figure 5:**
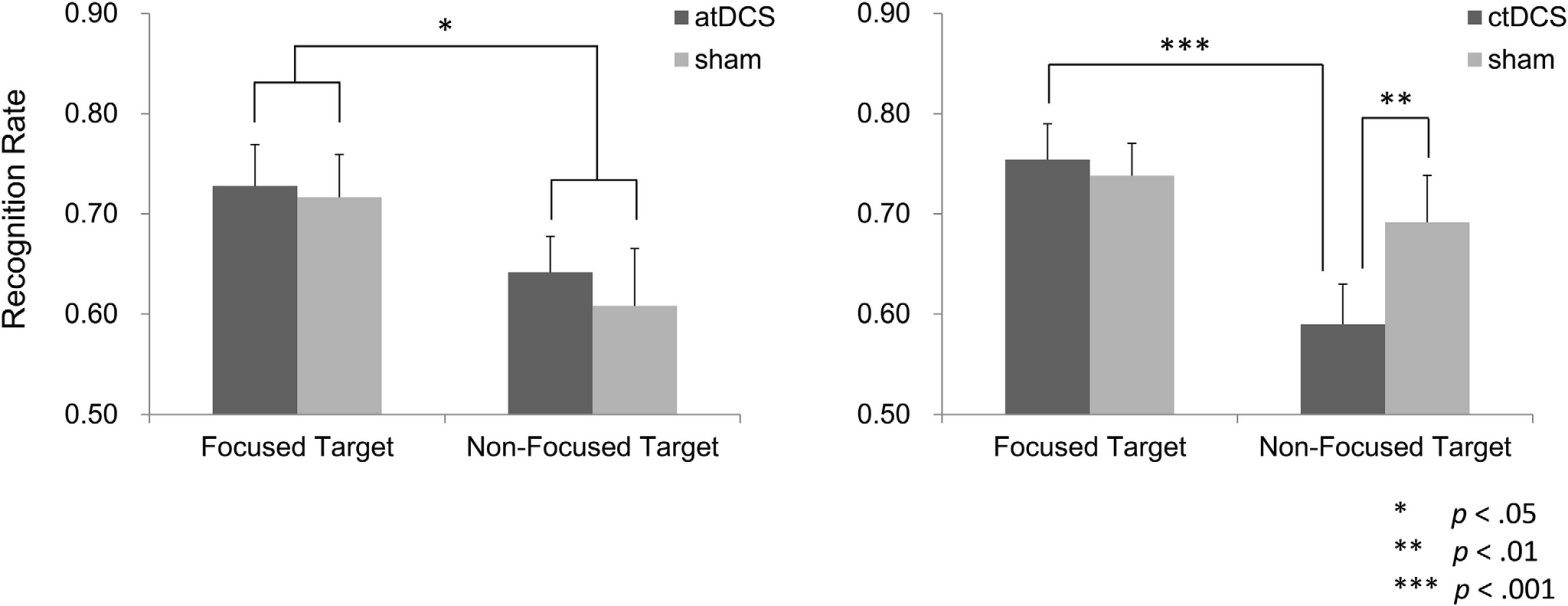
**Recognition performance of the target words**. In the atDCS group, recognition performance was higher in the target words in the focus condition (focused targets) than in the non-focus condition (non-focused targets), although brain stimulation did not modulate the performance. In the ctDCS group, however, recognition of the non-focus targets was lower under the ctDCS in comparison to the sham stimulation, while such difference was not observed in the focused targets.

In the atDCS group, recognition performance was better in the focus condition than in the non-focus condition (Figure 5). Administration of DC stimulation did not affect target recognition. A repeated ANOVA showed a significant main effect of the focus manipulation [*F*_(1, 15)_ = 6.38, *p* < 0.05], but not of brain stimulation, *F*_(1, 15)_ = 0.58, *p* > 0.05. There was no significant interaction between the factors [*F*_(1, 15)_ = 0.02, *p* > 0.05].

In the ctDCS group, target recognition for non-focus words was significantly lower under DC stimulation than sham (Figure 5). Such a difference was not observed for focus words. A repeated ANOVA yielded a significant main effect of the focus manipulation [*F*_(1, 15)_ = 19.29, *p* < 0.001] and a significant interaction between brain stimulation and focus manipulation [*F*_(1, 15)_ = 9.38, *p* < 0.01]. A main effect of the brain stimulation was insignificant [*F*_(1, 15)_ = 1.88, *p* > 0.05]. In *post-hoc* multiple comparisons, a significant simple main effect of the focus manipulation condition was observed in the ctDCS condition [*F*_(1, 15)_ = 26.78, *p* < 0.001], but not in the sham condition [*F*_(1, 15)_ = 2.49, *p* > 0.05]. Regarding brain stimulation, a simple main effect was found in the non-focus condition [*F*_(1, 15)_ = 7.50, *p* < 0.01], but not in the focus condition [*F*_(1, 15)_ = 0.18, *p* > 0.05]. Therefore, obtained interaction was attributed to the lower recognition performance for the non-focus target words under the ctDCS.

#### Distractor recognition

**Figure 6:**
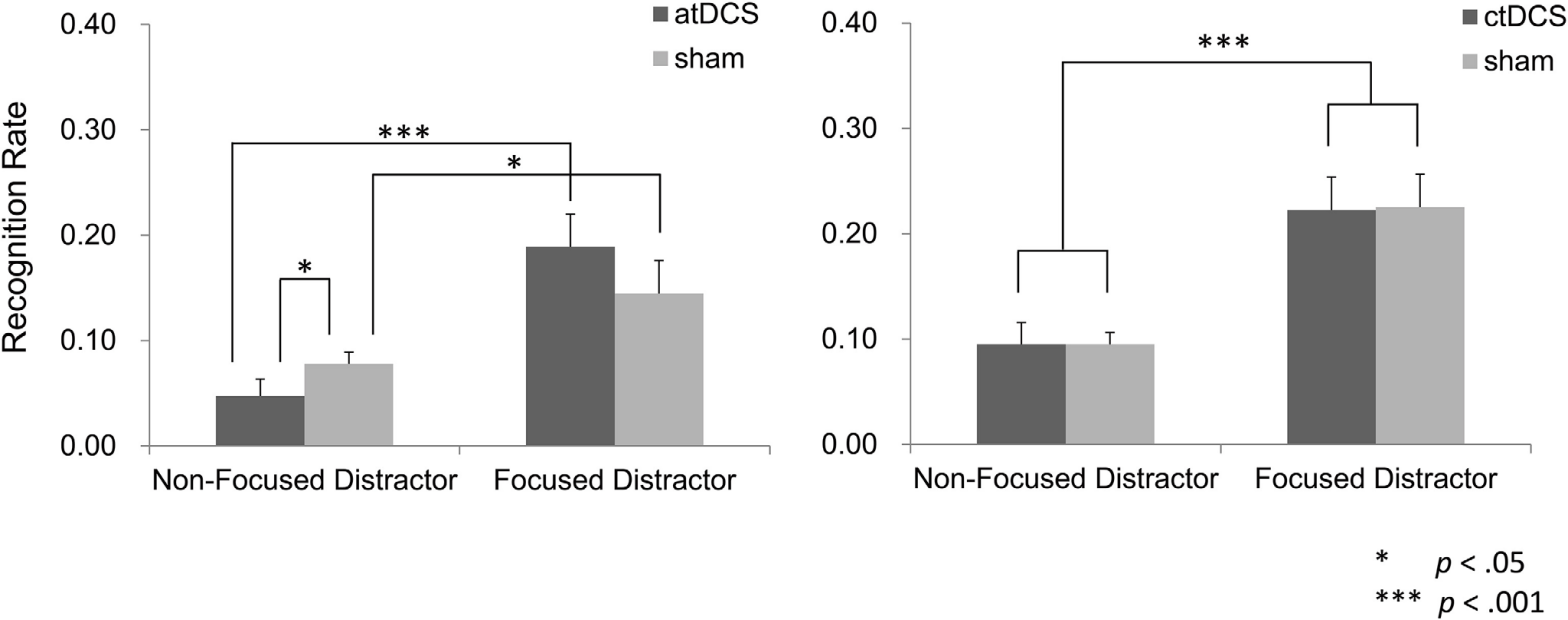
**Recognition performance of the distractor words. In the atDCS group, atDCS and sham stimulation tended to produce different effect across a type of distractor words**. Specifically, difference in recall performance between the non-focused distractor and the focused distractor was greater in the atDCS condition than in the sham condition. Such a recognition pattern was not observed in the sham condition.

In the atDCS group, we excluded data from one participant who showed an extremely deviated effect of brain stimulation on recognition of the focus distractor (2.9 *SD*s away from the mean) from the analysis. The effect of focus manipulation tended to be different between the DC and sham conditions. Specifically, difference in recognition performance between focused distractor words and non-focused ones were greater under atDCS than sham stimulation (Figure 6). A repeated ANOVA yielded a significant main effect of the focus manipulation [*F*_(1, 14)_ = 19.89, *p* < 0.001], but not of the brain stimulation [*F*_(1, 14)_ = 0.68, *p* > 0.05]. A trend toward significant interaction was obtained between factors of the focus manipulation and brain stimulation [*F*_(1, 14)_ = 4.30, *p* = 0.057]. A simple main effect analysis on the focus manipulation was found significant in both the DC [*F*_(1, 14)_ = 22.99, *p* < 0.001] and in sham conditions [*F*_(1, 14)_ = 5.09, *p* < 0.05]. A simple main effect of the brain stimulation was seen in the non-focused distractor words [*F*_(1, 14)_ = 4.62, *p* < 0.05], but not in the focused distractor words [*F*_(1, 14)_ = 2.00, *p* > 0.05]. Therefore, the obtain interaction is likely to be attributed to a greater effect of the focus manipulation under the atDCS than sham.

In the ctDCS group, recognition for focus distractors in the NF-RSTs was greater than that for non-focus distractors in the F-RSTs regardless of stimulation type (DC vs. sham) (Figure 6). A repeated ANOVA showed a main effect of the focus manipulation [*F*_(1, 15)_ = 30.58, *p* < 0.001], but not of the brain stimulation [*F*_(1, 15)_ < 0.01, *p* > 0.05]. There was no significant interaction [*F*_(1, 15)_ < 0.01, *p* > 0.05].

### Group analysis in the sham condition

As the focus effects in the recall and recognition tasks seem to be different between the atDCS and ctDCS groups in the sham stimulation, we analyzed the focus effect (i.e., absolute value of difference between performance in the focus condition and that in the non-focus condition) in each task measurement, using a between-subject *t*-test. As for the recall performance, the atDCS and ctDCS groups showed the equivalent focus effect under the sham stimulation, *t*_(30)_ = −0.74, *p* > 0.05. Similarly, in the intrusion error, focus effect did not differ between the groups, *t*_(30)_ = 0.73, *p* > 0.05. Regarding the target recognition, the focus effect did not differ between groups under the sham stimulation, *t*_(29)_ = 1.38, *p* > 0.05. A group difference was not found in the distractor recognition, either, *t*_(29)_ = −1.35, *p* > 0.05.

## Discussion

The present study investigated the effect of tDCS over the left PPC on attentional processing. Specifically, we examined whether brain stimulation modulates top-down attentional control or stimulus-driven attentional processing. We found that atDCS enhanced the effect of the focused word to augment differences in performance between the focus and non-focus conditions when compared with sham. Similarly, a *post-hoc* recognition task showed that difference in focused distractor words and non-focused ones were greater under the atDCS, supporting the idea that focused words attracted more attention when cortical excitability of the left PPC was enhanced. These results indicate that the atDCS enhanced stimulus-driven attentional processing in the verbal domain. As for ctDCS, RST performance did not differ with sham stimulation, but in the recognition task the non-focused target words were less recognized under ctDCS. This result may indicate that ctDCS impaired retrieval of less-important information from long-term memory, which is thought to require top-down attentional processing (Cabeza et al., 2008), or prevented less-important words from being transferred to long-term memory.

Although several studies have revealed the cognitive and neural mechanisms of visuospatial attention (e.g., Hopfinger et al., 2000; Corbetta and Shulman, 2002), far fewer have investigated verbal attention, which has taken a nature of the sentence into consideration. We have shown that a critical word in a sentence (focused word) is falsely recalled as a target word when presented as a distractor stimulus (Osaka et al., 2002b, 2007). This result indicates that the focused word attracts attention with its importance in reading comprehension. When contrasting individuals with high attentional control to those with low, number of false recalls was greater in the latter group (Osaka et al., 2002b), possibly because of lower inhibitory control for ignoring goal-irrelevant attractive distractors. Those results suggest that the present task requires two different kinds of PPC-related attentional processing: top-down attentional control and stimulus-driven attentional processing. Because atDCS enhanced cortical excitability to augment differences between the focus and non-focus conditions, stimulus-driven attentional processing is more likely promoted. atDCS directed attention toward the focused words in both conditions, resulting in better performance in the focus condition and poorer performance in the non-focus condition. Thus, atDCS over the left PPC, specifically P3 and surrounding areas, enhances the stimulus-driven attentional processing that depends on the inferior parietal cortex but not the top-down attentional processing that depends on the superior parietal cortex. This property may be explained by the electro-patch increasing the cortical excitability of the inferior parietal cortex or that neurons in the inferior parietal cortex might be more sensitive to atDCS. atDCS did not affect intrusion errors (false recall of distractor words), possibly because of the small number of trials. However, in the recognition task, participants again showed a greater effect of the focus manipulation under the atDCS, reflecting attentional capture by the focused words during RSTs. Taken together, the cortical excitation promoted by atDCS enhanced stimulus-driven attentional processing.

Contrary to our expectation, ctDCS did not produce the opposite effect of atDCS on RST performance. Such a heterogeneous effect by tDCS has been described previously (Jacobson et al., 2012). The null effect of ctDCS can be attributed to higher activation of the PPC while performing RSTs (Silvanto et al., 2008). However, in the recognition task, target recall of the non-focus words was significantly reduced compared with shams. According to Cabeza et al. (2008), episodic retrieval can be divided into two parallel streams of attentional processing. They proposed top-down memory retrieval that depends on the superior parietal cortex and less demanding retrieval with high confidence that relies on the inferior parietal cortex. Therefore, less recognition of the unfocused target words may be due to an impaired superior parietal cortex, because retrieval of less important words would require a more strategic search in episodic memory. Another possibility is that ctDCS over the left PPC prevented less important information from being transferred to long-term memory. However, this might be less likely as few studies have suggested the involvement of the PPC in memory consolidation.

The present study has several limitations; therefore, one must regard the conclusion as tentative and further elaborated work is mandatory. The first limitation is a possible placebo effect of the tDCS in which perception of electro-stimulation (2 mA) could affect cognitive performance as shown in the previous study (Ambrus et al., 2010). Although most of the participants did not report continuous perception of electro-stimulation, possibly due to an alleviative effect on pain perception by the highly attention-demanding task (Veldhuijzen et al., 2006), subjective itch or pain scale needed to be collected to eliminate a possibility of the placebo effect. The second limitation is an ambiguity of a focal effect of the tDCS. Unless we perform simultaneous measurement of brain activity and brain stimulation, it is hard to tell whether a stimulated region is really activated or suppressed, as suggested in the previous literature (Bai et al., 2014). The third limitation is a variability of a focus effect across participants in the present task. Our previous study has shown that individuals with low working memory capacity were more susceptible to distraction by a focus distractor word; therefore, they showed greater focus effect (Osaka et al., 2002a). As described in the method section, we measured individual working memory capacity prior to the tDCS experiment and confirmed that means of working memory capacity did not differ between the atDCS group and ctDCS group. Furthermore, when compared recall and recognition performance between the groups in the sham condition, all the behavioral indices did not statistically differ between groups (*p* > 0.05). However, the focus effect in the sham conditions seems to be slightly different between the atDCS and ctDCS group. In fact, multiple-comparisons analysis showed that focus effect (difference between the focus condition and the non-focus condition) in the sham condition was significant in the ctDCS group while the effect was a significant trend in the atDCS group. To avoid such individual variability of the focus effect, a future study needs to perform an inter-subject design, where each participant received anodal, cathodal, and sham stimulation. The forth limitation, which is the most critical, is lack of a polarity effect of the DCS. Although we mentioned that our primary goal was to test an effect of the tDCS in comparison to the sham stimulation, one needs to obtain a main effect of the polarity or interactions between the polarity of DCS and the other experimental factors to argue that the enhancement of the focus effect was anodal-specific or cathodal-specific. When analyzing data including a factor of the polarity of DCS, none of the statistical analysis showed a significant main effect of the polarity and interactions between the polarity effect and other factors (i.e., focus/non-focus manipulation and DCS/sham stimulation) (*p* > 0.05). Similar issue might be found in previous studies in the field of working memory and attention. For instance, Fregni et al. (2005) have found that atDCS over the left dorsolateral prefrontal cortex enhanced working memory. In the study, they suggested the polarity effect of the atDCS by presenting a result of equivalent effect between the sham and cathodal stimulation, but not directly comparing effects between the atDCS and ctDCS stimulation. As discussed above, tDCS may produce a heterogeneous effect in the field of cognition (i.e., enhancement effect of the atDCS but no effect of the ctDCS), which makes it harder to detect a statistically significant polarity effect. However, such issue has to be resolved by specifying an effective protocol of stimulation (intensity, frequency, schedule, and so on). Finally, the present study employed an online stimulation which temporally modulated brain activity to examine the function of a specific brain area. However, considering the therapeutic use of the tDCS that aims to promote recovery of brain functions or delay progress of disease, we need to test a continuous effect of the tDCS on focus effect using an offline method and specify the most effective stimulation protocol, which might assist people who have difficulty in reading comprehension by directing their attention toward the critical word of a sentence.

## Conflict of interest statement

The authors declare that the research was conducted in the absence of any commercial or financial relationships that could be construed as a potential conflict of interest.
