# Supplementary material for: The anodal tDCS over the left posterior parietal cortex enhances attention toward a focus word in a sentence
Source: Front Hum Neurosci. 2014 Dec 9;8:992. doi: 10.3389/fnhum.2014.00992 (PMC4260498; doi:10.3389/fnhum.2014.00992)
Supplement: Supplementary file 1 [file Image1.PDF]

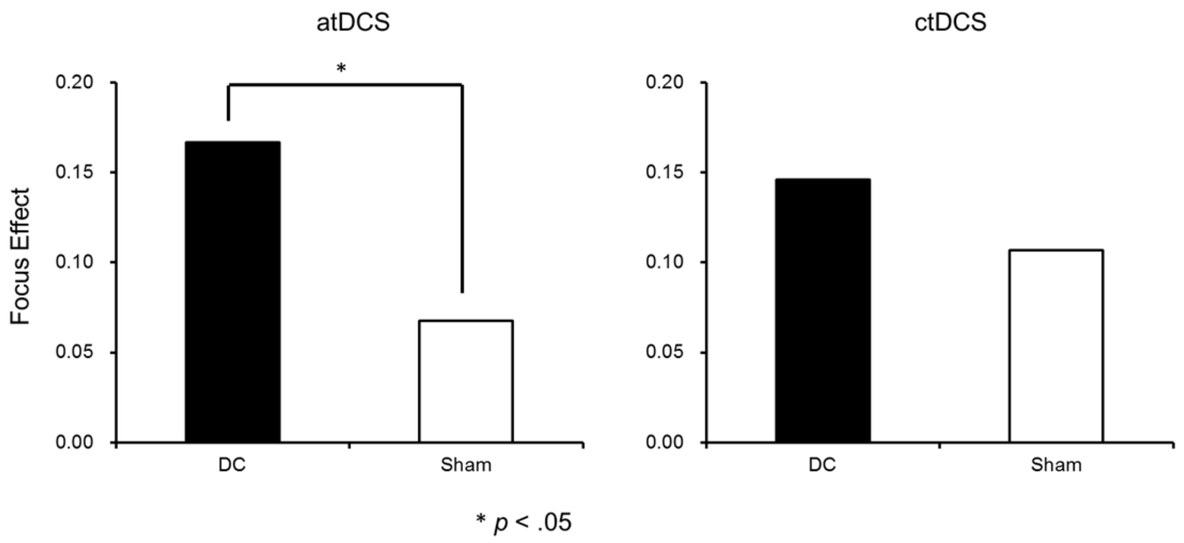

Supplementary figure 1. The focus effect in the recall performance under the brain stimulation. In the atDCS group, the focus effect was greater in the DC condition than the sham condition. In the ctDCS group, the focus effect did not differ between conditions.

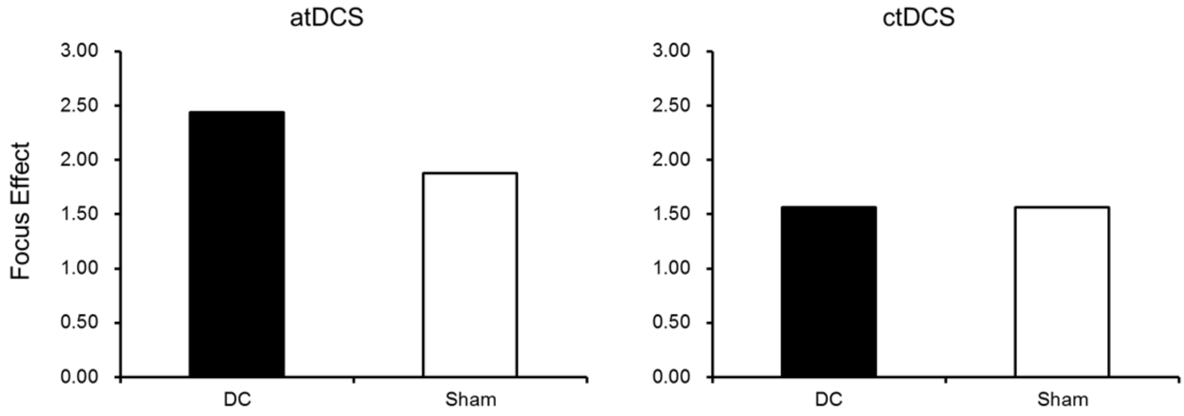

Supplementary figure 2. The focus effect in the intrusion error under the brain stimulation. In both groups, the focus effects did not differ between the brain stimulation conditions.

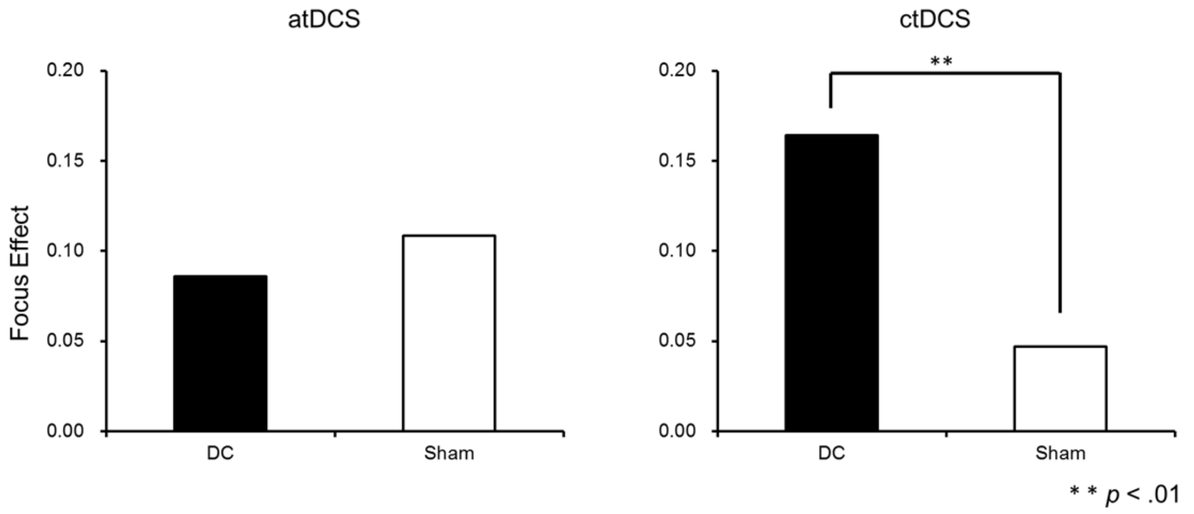

Supplementary figure 3. The focus effect in the target recognition under the brain stimulation. In the atDCS group, the focus effect did not differ between the brain stimulation conditions. In the ctDCS group, on the other hand, the focus effect was greater under the DC stimulation condition than the sham condition.

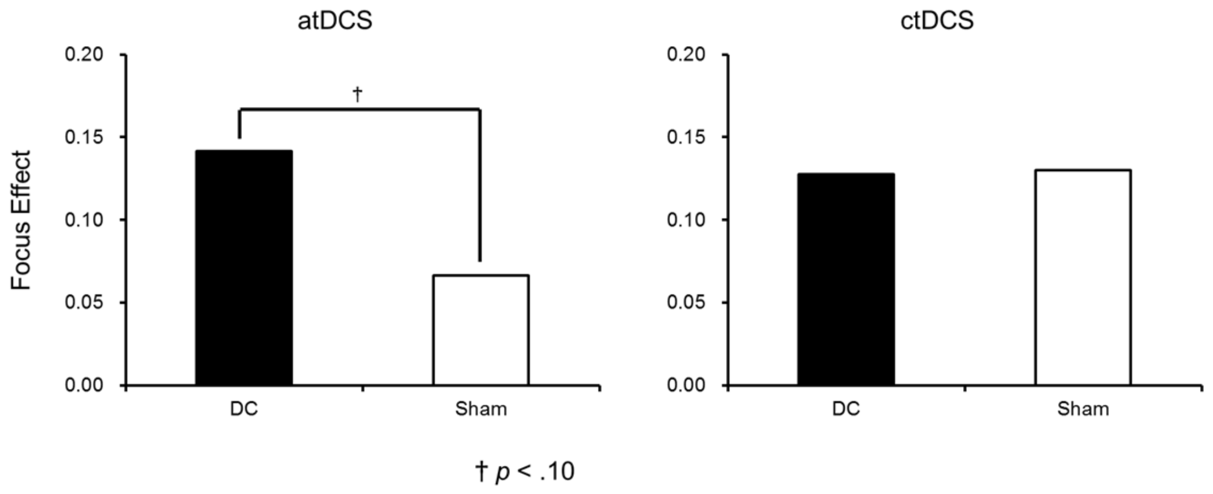

Supplementary figure 4. The focus effect in the distractor recognition under the brain stimulation. In the atDCS group, the focus effect tended to be greater in the DC condition than the sham condition. In the ctDCS group, the focus effect did not differ between conditions.
